# Geminin Orchestrates Somite Formation by Regulating Fgf8 and Notch Signaling

**DOI:** 10.1155/2018/6543196

**Published:** 2018-06-07

**Authors:** Wei Huang, Yu Zhang, Kang Cao, Lingfei Luo, Sizhou Huang

**Affiliations:** ^1^Key Laboratory of Freshwater Fish Reproduction and Development, Ministry of Education, Laboratory of Molecular Developmental Biology, School of Life Sciences, Southwest University, Beibei, Chongqing 400715, China; ^2^Development and Regeneration Key Laboratory of Sichuan Province, Department of Anatomy and Histology and Embryology, School of Basic Medicine, Chengdu Medical College, Xindu, Chengdu 610500, China; ^3^Department of Pathogen Biology, School of Basic Medicine, Chengdu Medical College, Xindu, Chengdu 610500, China

## Abstract

During somitogenesis, Fgf8 maintains the predifferentiation stage of presomitic mesoderm (PSM) cells and its retraction gives a cue for somite formation. Delta/Notch initiates the expression of oscillation genes in the tail bud and subsequently contributes to somite formation in a periodic way. Whether there exists a critical factor coordinating Fgf8 and Notch signaling pathways is largely unknown. Here, we demonstrate that the loss of function of geminin gave rise to narrower somites as a result of derepressed Fgf8 gradient in the PSM and tail bud. Furthermore, in geminin morphants, the somite boundary could not form properly but the oscillation of cyclic genes was normal, displaying the blurry somitic boundary and disturbed somite polarity along the AP axis. In mechanism, these manifestations were mediated by the disrupted association of the geminin/Brg1 complex with intron 3 of mib1. The latter interaction was found to positively regulate mib1 transcription, Notch activity, and sequential somite segmentation during somitogenesis. In addition, geminin was also shown to regulate the expression of deltaD in mib1-independent way. Collectively, our data for the first time demonstrate that geminin regulates Fgf8 and Notch signaling to regulate somite segmentation during somitogenesis.

## 1. Introduction

Somitogenesis is a critical developmental event whereby pairs of epithelial spheres, named somites, form periodically from the mesenchymal presomitic mesoderm (PSM) [1]. The “clock and wavefront” model was proposed to explain the mechanism of somite formation [2–4]. This model postulates interactions between the wavefront of gradients (e.g., those of Fgf8) and the segmentation clock (cyclic genes) in the PSM that gate cells into potential somites [3]. Many studies in different animals identified a kind of molecular oscillators named “segmentation clock,” which gives rise to oscillations of gene expression levels in the PSM. The stabilization of the oscillations in the anterior PSM leads to the establishment of segment polarity and sequential morphological segmentation [5].

In mouse, the segmentation is disorganized in embryos with mutations of* Notch1* [6],* Su(H)/RBPJj* [7], and other Notch-related genes [8–10]. It was also reported that in Xenopus and zebrafish, either dysregulation of the ubiquitous activation of Notch signaling or its inhibition by injections of protein-encoding* mRNAs* caused aberrant somite formation [11–13]. In addition, mutants with mutations in Notch signaling-related genes also displayed segmentation defects [14, 15], including disrupted somite boundary formation [14–17] and “salt and pepper” expression pattern of cycling genes such as* deltaC* and* her1* [18, 19]. These reports suggested that tight control of Notch signaling was crucial for proper somite segmentation.

Expression of Fgf8 in the tail bud and PSM changes from low to high expression level along the anterior-posterior (AP) axis [20–24], working as the wavefront molecular cue to gate anterior PSM cells into newly forming somite [21, 23, 24]. An increase in the local concentration of the Fgf8 protein in the PSM reduced somite size, whereas Fgf signaling inhibition induced the formation of larger somites [21, 24]. Besides the role of FGF signaling in somite size control, early studies in mouse suggested that FGF signaling acts upstream of Notch and Wnt signaling pathways [25]. However, this epistatic relationship is not clear, because it was also reported that Wnt lies upstream of Notch and FGF [26–28]. In addition, microarray studies of mouse PSM transcriptome showed that the downstream genes* Spry2* and* Dusp6 *of the FGF signaling pathway are expressed in the PSM with a cyclic expression pattern in a Notch-independent way [29], further suggesting a complex network of FGF, Notch, and Wnt signaling pathways [29] and a complicated mechanism of how these three signals are orchestrated during somitogenesis. In zebrafish, although* her13.2* was reported to link Fgf signaling to the Notch-regulated oscillation machinery [30], whether there exists an upstream factor that coordinates Notch and Fgf signaling and thereby orchestrates somite segmentation is still unknown.

Geminin, in addition to its well-known role in regulating cell cycle [31–33], is involved in the regulation of neuronal development, hematopoiesis, and stem cell maintenance [34–41]. In zebrafish, geminin also plays a critical role in gastrulation cell movement, eye development, and left-right (LR) patterning [42–44]. The geminin gene is expressed ubiquitously before the gastrulation stage. From the early somite stage, it is expressed in the tail bud, PSM, and newly formed somite [42, 44] (Figure S1 A). The expression pattern of geminin at the somite stage is similar to that of* fgf8*, which serves as the posterior wavefront gradient regulating segmentation position in the lateral plate mesoderm. These observations prompted us to hypothesize that geminin is involved in regulating somitogenesis in early zebrafish development. Here, we found that geminin loss of function led to blurry somitic boundary and smaller somite. We propose that geminin simultaneously regulates Fgf8 and Notch signaling and shapes somite boundary and somite size during somitogenesis.

## 2. Materials and Methods

### 2.1. Zebrafish Strain and Maintenance

Zebrafish (Danio rerio) of the AB genetic background was maintained, raised, and staged as described previously [45]. The transgenic fish line* Tg (hsp70l:dnfgfr1-EGFP)* used in this study is gift from Didier Y. R. Stainier Lab.

### 2.2. Morpholinos and* mRNA* Injection

Antisense ATG morpholinos (Gene Tools) against both maternal and zygotic* geminin *(*GemMO*, 5′-CTTTGGTCTTCTGATGGAACTCATA-3′) [42],* p53 *(*p53MO*, 5′-GCGCCATTGCTTTGCAAGAATTG-3′) [46],* Mib1 *(*Mib1MO*, 5′-GCAGCCTCACCTGTAGGCGCACTGT-3′) [47],* Brg1 *(*Brg1MO*, 5′-CATGGGTGGGTCAGGAGTGGACATC-3′[48]),* fgf8 *(*fgf8MO*, 5′-TGAGTCTCATGTTTATAGCCTCAGT-3′) [49], and control morpholino (conMO, 5′-CCTCTTACCTCAGTTA CAATTTATA-3′) [42] were injected into the yolk of one-cell stage embryo. The following concentration was used: GemMO (5ng), p53MO (2ng), Mib1MO (3ng), Brg1MO (1ng), Fgf8MO (1ng), and control MO (5ng).* Geminin mRNA*,* fgf8 mRNA, GFP mRNA*, and* NICD mRNA *were synthesized in vitro according to the manual of Kits (Ambion). In the rescue experiments,* geminin mRNA (15pg)* and* NICD mRNA (2pg)* were injected into the yolk at 1-cell stage. For overexpression of* fgf8* and* NICD* in one-half of the embryos, the* GFP mRNA (10pg)*,* NICD mRNA (15pg), *and* fgf8 mRNA (15pg)* were injected into one cell at 4-8 cells' stage.

### 2.3. Chemical Treatment and Heat Shot Treated for Embryos

Zebrafish embryos were incubated with 0.4 uM BMS453 (0.8 ul of 10mM BMS453 stock diluted in 20 ml of egg water) [44]. Treated embryos were washed twice and cultured in egg water until fixation or observation. In all experiments, treated embryos were compared with mock treated control siblings (0.8 ul of DMSO diluted in 20 ml of egg water). To block the Notch activity, the embryos were incubated with DAPT (50 *μ*M) [50] dissolved in 5 mL of egg water; 0.1% DMSO was used as a negative control. Heat shock treatment for transgenic line* Tg (hsp70l:dnfgfr1-EGFP)*: Embryos were cultured at 28.5°C to 40% epiboly and then put at 39°C for 40 minutes and returned back to incubator at 28.5°C. The embryos were screened for positive and negative GFP for fixation and observation at stages needed.

### 2.4. Whole Mount In Situ Hybridization, TUNEL Assay, and Cell Transplantation

Whole mount in situ hybridization was performed as previously described [51], using established antisense probes. The following digoxigenin-labeled antisense probes were used:* delta C*,* delta D*,* fgf8*,* her1*,* her4*,* her7*,* mib1*,* raldhl2*,* mespaa*,* mespba*,* papc*, and* tbx16l*. The TUNEL assay was performed using an in situ Cell Death Detection kit (Roche) as described by the manufacturer [42]. Cell transplantation was performed as described previously [42]; at 1000- to 2000-cell stage, 40-70 cells in the margin region from donor embryos, coinjected with* geminin MO* and Dextran-Alexa 568, were transplanted into the control embryos; then the embryos were screened out; only half of the embryos that owned Dextran-Alexa 568 were observed and fixed.

### 2.5. ChIP Experiments and Quantity-PCR

Chromatin immunoprecipitation (ChIP) was done according to standard protocol. 21 somite stage zebrafish embryos (AB strain) were used to perform ChIP by using anti-geminin antibody or normal rabbit serum (NRS). Briefly, for each immunoprecipitation, embryos were dechorionated and fixed in 1% formaldehyde in 1X embryo medium for 20 min at room temperature. Fixed embryos were homogenized in lysis buffer and incubated for 20 min on ice. Nuclei were collected by centrifugation, resuspended in nuclei lysis buffer, and then incubated for 10 min before diluting with IP buffer and sonicating the chromatin sample on an ice bath. The lysate was incubated overnight at 4°C with protein A/G Agarose/Salmon Sperm DNA prebound to the antibody. Beads were washed with Low Salt Immune Complex Wash Buffer (Catalog # 20-154, Upstate), High Salt Immune Complex Wash Buffer (Catalog # 20-155, Upstate), LiCl Immune Complex Wash Buffer (Catalog # 20-156, Upstate), and TE buffer (Catalog # 20-157) successively and then eluted at 65°C in elution buffer and cross links were reversed. Chromatin was purified by treatment with RNase A, followed by proteinase K digestion and extraction. For sequencing the purified chromatin, blunt-ended DNA fragments for cloning purposes were created by T4 DNA polymerase. After ligating the samples into pCRII-TOPO plasmid at 16°C overnight, we transform the ligation mixture into competent bacterial cells. During 37°C incubation, we inoculate the number of colonies and miniprep PCR positive clones and sent them to sequence. The sequencing data were blasted and analyzed with NCBI database. For qPCR, we prepared a PCR mix and aliquot for individual 25ul PCR reactions for all ChIP and input samples. In the qPCR, the primers for partial intron 3 of Mib1 were as follows: Mib1 intron 3_5: ggtcaaggtgctccaggattg; Mib1 intron 3_3: gtgactgtatttgatgtctctgtt. Prepare and establish a standard curve for these primers and calculate the amount of DNA in each sample. Determine the amount of precipitated DNA relative to input as [(amount of ChIP DNA)/ (amount of input DNA)] x100.

### 2.6. Luciferase Report Analysis

Partial intron 3 of Mib1 was amplified by PCR from genomic DNA prepared from AB strain zebrafish, cloned into pCR-TOPO II vector, and subcloned into pGL3-promoter vector (Promega). Luciferase Assay was carried out according to the manuscript (Promega). One-cell embryos were injected with 40pg luciferase constructs, with or without* GemMO *(5ng),* Brg1MO *(4ng), wild-type* Brg1 mRNA* expressed construction (5ng), or* geminin mRNA *(30pg). At 10th somite stage, 30 to 50 embryos were collected and homogenized and samples were prepared. Samples were then diluted 5-10-fold and quantified using the Dual Luciferase Assay kit (Promega). Each experiment was performed 3 times minimum. All data are reported as the mean fold change in luciferase activity compared to the condition where no mRNA or no morpholinos were injected and reported with standard error of the mean. Differences in the luciferase activity for different samples were compared by T-test. P value 0.05 was considered significant.

### 2.7. Microscopy

Whole mount in situ hybridized larvae were imaged using a SteREO Discovery V20 microscope equipped with AxioVision Rel 4.8.2 software (Carl Zeiss, Jena, Germany) [52–54].

## 3. Results

### 3.1. Geminin Regulates Somite Formation in the Early Development

**Figure 1:**
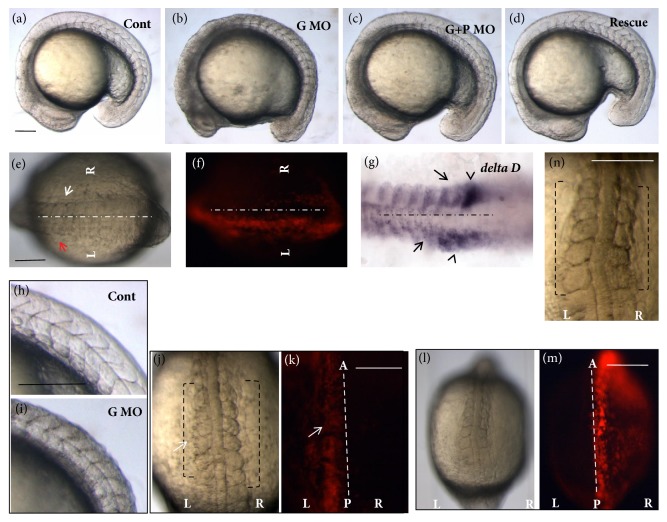
**Geminin regulates somite formation during early embryogenesis**. (a-d) Compared with that in control morphants (a, n=24), the somite boundary is vague in* geminin* morphants (b, 86.4%, n=43) as well as in* geminin* and* p53* double morphants (c, 84.3%, n=51). Meanwhile, the somite phenotype in geminin morphants was partially rescued by injection of* geminin mRNA* (d, 63.3%, n=49). (e-g) The cells downregulated the role of geminin which was transplanted into left side of the embryos (f, n=3); the somitic boundary in the cell transplanted side was vague (e, left side, arrow head shown, n=3).* DeltaD *in situ staining for transplanted side (g, left side) and nontranslated side (g, right side) indicated decreased expression of* deltaD* (g, black arrow) and defective somite boundary formation (g, black arrow head). (h-k) Somite space was narrow in* geminin* morphants (i, 80.4%, n=46) when compared with that in control morphants (h, 100%, n=18). In the transplanted embryos, the transplanted side (GemMO injection) of the embryos (j, k, left side, white arrow shown) displayed smaller somite when compared with that in control side ((j, k, left side, white arrow shown). Meanwhile, when GemMO and p53MO were coinjected into the donor embryos, the transplanted side of the embryos also displayed smaller and vague somite (l-n). L, left side; R, right side. Bar, 100*μ*M.

The expression pattern of the geminin gene (Figure S1 A) suggests a possible role of geminin in somitogenesis. To confirm this, we synthesized geminin MO* (GemMO)* to block geminin translation [42, 44] (Figure S1 B). When geminin was knocked down, the embryos did not show any obvious defect except for the shortened AP axis during gastrulation (Figure S1 C). At the early somite stage, the embryos displayed developmental delay, cell apoptosis, deformed somite shape, and blurry somitic boundary (Figures 1(a), 1(b), and S1 D). Geminin is a crucial cell cycle regulator and geminin loss of function* in vivo* has been shown to cause cell cycle defect and subsequent cell apoptosis [42]. To check whether the defective somite phenotype resulted from cell apoptosis in* geminin* morphants, we coinjected* GemMO* and* p53MO* together [44, 55] and examined whether defective somites existed in* Gem/p53* morphants. The embryos injected with* GemMO* and* p53MO* did not exhibit developmental delay but displayed disturbed somites as in geminin morphants (Figure 1(c)). We further employed another two methods to confirm the specific role of geminin in somitogenesis. First, we coinjected* GemMO* and* GemMO-*resistant geminin* mRNA* into the embryos and found that the geminin* mRNA* rescued the somite phenotype caused by geminin loss of function (Figure 1(d)). Secondly, at the dome stage, we transplanted the cells from the donor embryos injected with* GemMO* into the wild-type embryos. At the 10th somite stage, we assessed the somite phenotype and found that only half of embryos showed transplanted cells (Figure 1(f)). Moreover, the left side of the embryos, into which donor cells were transplanted, developed slowly and exhibited blurry somite boundary (Figures 1(e) and 1(f), red arrow) when compared with that in the control right side (Figures 1(e) and 1(f), white arrow). This result was consistent with that of in situ staining for* deltaD *probe, in which the newly formed somite boundary was not clear (Figure 1(g), arrow) and* deltaD* expression in forming somites was delayed (Figure 1(g), arrow head). We also transplanted* GemMO* and* p53MO* double knockdown cells into the wild-type embryos (Figures 1(l) and 1(m)) and found that the somite boundary was not clear (Figure 1(n)). These findings suggested that geminin loss of function led to somitic segmentation defect during somitogenesis. Furthermore, detailed analysis revealed that the somitic spaces along the AP axis became smaller in geminin morphants than in control morphants (Figures 1(h) and 1(i)). In addition, we also found that the transplanted side of embryos exhibited narrower somites when compared with those on control side (Figures 1(j) and 1(n), brackets). These data showed that geminin regulates not only the formation of the proper somitic boundary, but also somite space patterning along the AP axis during somitogenesis.

### 3.2. Antagonist Gradient between Fgf8 and Retinoic Acid (RA) Affects the Role of Geminin in Somite Size Patterning

**Figure 2:**
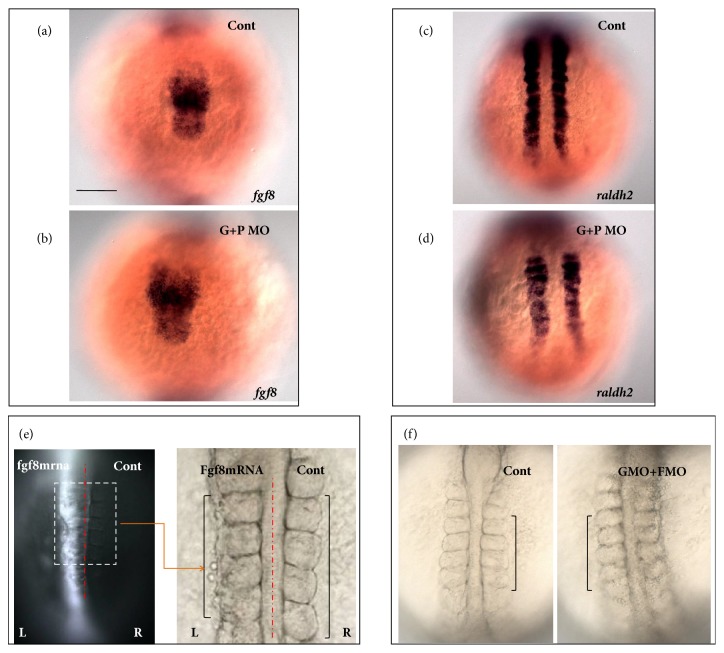
**Fgf8 and RA signaling mediate geminin to regulate proper somite space**. (a-d) When compared with that in control (a, c)* fgf8* expression was upregulated in* geminin *and* p53 *double morphants (b, 86%, n=28), but the expression of* raldh2* was downregulated in* geminin* morphants (d, 81%, n=26). The embryos dominantly expressed GFP and Fgf8 in the left side (e, n=5); overexpression of Fgf8 in the left side of the embryos gave rise to narrow somite (e, left side, 100%, n=5). In the embryos coinjected with* geminin MO* and* fgf8 MO* (f, right side, 77%, n=22), the somite size is close to that in control embryos. Bar, 100*μ*M.

Fgf8 plays an important role in maintaining PSM cell fate [22, 24]. Transplantation of Fgf8 beads into PSM regions maintains their posterior axis and gives rise to shorter somites [21, 24]. In embryos injected with* GemMO*, the somite length along the AP axis became shorter (Figure 1). Thus, we examined the expression of* fgf8* in geminin morphants as well as geminin and p53 double morphants and found that it was increased in both the PSM and tail bud (Figure 2(b) and Figure S2 A, B).* Tbx16l*, a downstream gene in the Fgf8 pathway, determines the formation of the posterior axis in the PSM [56]. Our experimental results revealed that the expression of* tbx16l* was also significantly increased in geminin morphants (Figure S2 E, F). RA and Fgf8 have been reported to serve as antagonistic gradients (also shown in Figure S3) that control somite symmetric patterning and segmentation boundary formation [12, 57]. We also examined the role of geminin in RA expression. In the 10th somite stage wild-type embryos,* raldh2* was expressed in the heart progenitor field (Figure S2 C, arrow head), somite, and the anterior part of the PSM area (Figure S2 C) and formed a gradient opposite to that of* fgf8*. In geminin morphants,* raldh2* was significantly downregulated in the heart progenitor field (Figure S2D, arrow head), somite region, and the anterior part of the PSM (Figure S2D). In geminin and p53 double morphants, we also found the raldh2 was downregulated (Figure 2(d)). These data demonstrate that geminin likely regulates the proper somite size via controlling the concentration gradient of Fgf8 and RA in the posterior and anterior part of the PSM during somitogenesis. To evaluate this hypothesis, first we overexpressed* fgf8 mRNA* in the left side of the embryos (Figure 2(e), right embryos) and found that this manipulation led to shorter somites in the left PSM (Figure 2(e), left bracket) when compared to those on the right side (Figure 2(e), right bracket). These results were consistent with those in an earlier report in zebrafish [24]. In addition, we examined if the downregulation of Fgf8 signaling in geminin morphants increased the somite size by coinjecting* GemMO* and* Fgf8 MO*. Somite size in embryos injected with both MOs was similar to that in control embryos (Figure 2(f), shown by the left and right brackets). The results above suggested that, at least partially, the antagonist gradients of Fgf8 and RA signaling affect how geminin regulates proper somite size patterning during somitogenesis.

### 3.3. Role of Notch Signaling in Mediating the Effects of Geminin Loss of Function

**Figure 3:**
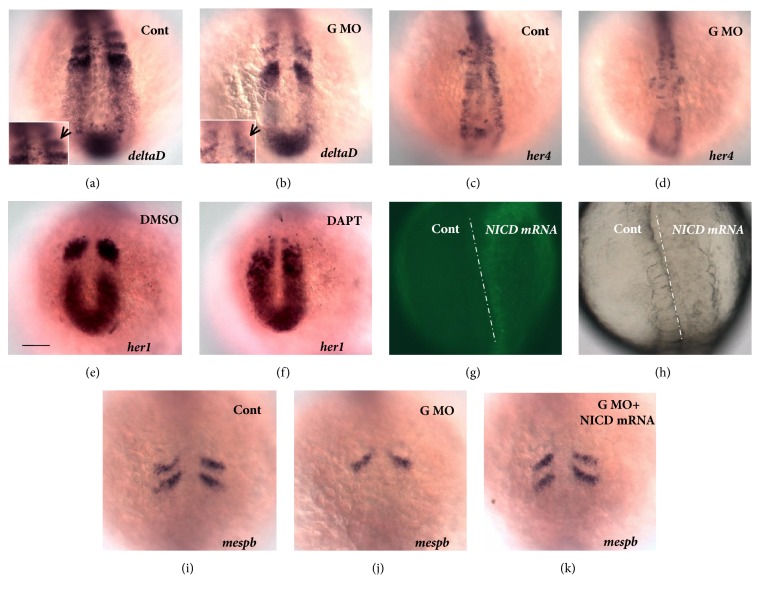
**Notch activity in geminin morphants and the role of Notch in somitogenesis**. (a, b) To compare with the expression of* DeltaD* in control (a, 88.5%, n=35),* deltaD* was downregulated in* geminin* morphants (b, 82.2%, n=45). The stripes of* deltaD* in new forming somite displayed wild “salt and pepper” way (a, b, arrow showed). (c, d) The downstream gene of Notch signal* her4* was also downregulated in the embryos injected with* GemMO* (d, 85.7%, 49), while the expression of* her4* was normal in control embryos (c, 82.8%, n=35). (e, f) The cyclic gene* her1* was normal in the embryos treated with DMSO (e, 100%, n=22); the oscillation of* her1* was blocked in the embryos treated with DAPT (f, 100%, n=18). Overexpression of NICD in the right side of the embryos (g, right side) resulted in disrupted somite segmentation (h, right side). (i-k) Comparing with that in control (i, 86.3%, n=22) and* geminin* morphants (j, 71.4%, n=28), the expression pattern and quantity were partially restored by coinjecting low dose of* NICD mRNA* (k, 53.2%, n=32). Bar, 100*μ*M.

Somitogenesis disturbance caused by Notch signaling deficiency has been demonstrated in many species in the animal kingdom [3, 5, 11, 15, 16, 35]. In zebrafish, the Notch ligand DeltaD initiates the segmentation clock and induces the start of oscillations in the expression of several genes [57]. DeltaC, another Notch ligand, maintains and promotes the coordinated expression of the oscillator [57]. After* GemMO *injection, somite edges and spacing became blurred (Figures 1(b), 1(i); S1F, E). Our results further demonstrated that the expressions of* deltaD* (Figure 3(b)) and the downstream gene of Notch signaling* her4* (Figure 3(d)) were decreased by loss of function of geminin. Although the expression levels of* deltaC *(Figure S4B) as well as the oscillators* her1* (Figure S4D) and* her7* (Figure S4F) were slightly downregulated, their expression patterns were not changed. Furthermore, the stripe of* deltaC* in newly formed somite exhibited a weak “salt and pepper” pattern (Figure S4B, shown by arrow).

The transcription factors of the mouse Mesp family were shown to act upstream of a genetic cascade involving the Notch pathway; they suppress Notch activity, which ultimately results in boundary positioning and the formation of anterior and posterior somatic compartments [58, 59]. In zebrafish, the expression of* mesp* family genes was decreased in segmentation defect embryos [60]. Overexpression of* mesp/mespaa* downregulated the expression of* notch5* and led to defective somitogenesis [61]. In* mesp* quadruple mutant embryos, somite formation was abnormal, and each somite was disrupted in a manner similar to that of the mouse* Mesp2* mutant [62]. We found that the expression levels of* mespba* (Figure 3(j)),* mespaa* (Figure S3H), and another somite anterior polarity regulator* papc* (Figure S4J) were reduced in geminin morphants. These data suggested a possibility that geminin regulates somitogenesis and somite anterior polarity by controlling the activity of Notch. To study if Notch signaling is regulated by geminin in this process, we analyzed the role of Notch during somitogenesis. Blocking Notch signaling in the embryos by incubating them with the *γ*-secretase inhibitor N-[N-(3,5-difluorophenacetyl)-L-alanyl]-S-phenylglycine* t*-butyl ester (DAPT) [63, 64] led to defective somite formation (data not shown) and the loss of cyclic expression pattern for* her1* (Figure 3(f)). Consistent with an earlier report [50], overexpression of Notch intracellular domain (NICD)* mRNA* in the right half of the examined embryos (Figure 3(g), right side) also induced deformed somites (Figure 3(h), right side). Furthermore, a low dose of NICD* mRNA* partially rescued the disrupted expression pattern of* mespba *(Figure 3(k)), showing that 53.2% of the embryos injected with both* GemMO* and NICD* mRNA* displayed two stripes of* mespba* in the anterior PSM, whereas only 28.6% of embryos injected with* GemMO* displayed two stripes of* mespba *(Figure 3(j)). Thus, all these data suggested that* GemMO* downregulated Notch signaling and did not lead to dysregulation of the oscillation gene expression. Therefore, Notch activity partially mediates the role of geminin in proper somite formation.

### 3.4. Regulation of mib1 Expression by Geminin

**Figure 4:**
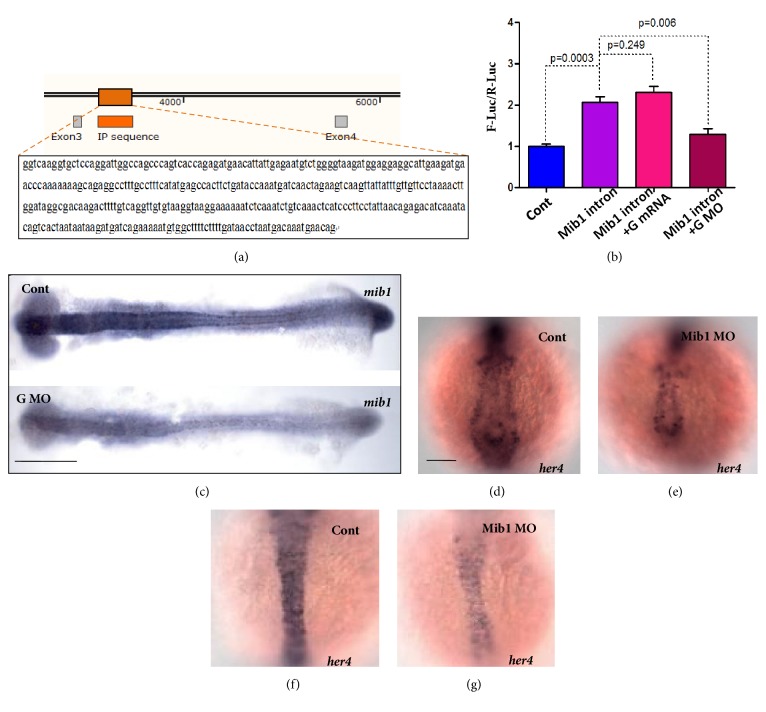
**Geminin positively regulates the transcription of Mib1 by associating with the intron 3 of Mib1**. (a, b) Partial intron 3 of Mib1 was identified to be associated with geminin by ChIP cloning experiment (a). Luciferase report analysis revealed that the partial intron 3 of Mib1 works as enhancer to regulate Mib transcription (b, line 2), and geminin positively regulates the transcription of* Mib1 *(b, line 3 and line 4). (c) To compare with the expression of* mib1* in control (c, upside, 87.8%, n=41),* mib1* was downregulated in* geminin* morphants (c, downside, 84.8%, n=46). (d-g) Comparing with that in control (d, f, 82.8%, n=34,) the activity of Notch signaling was downregulated greatly in* Mib* morphants (e.g., 84.4%, n=32), showing decreased expression of* her4* in the PSM, tail bud, the forming somite (e), and midline (g). Bar, 100*μ*M.

Geminin has been reported to be involved in the regulation of gene transcription by binding to the gene regulating elements within the regulatory protein complexes during neurogenesis and hematopoiesis [35, 41, 65–67]. To reveal how geminin regulates Notch activity, we prepared an antibody against zebrafish geminin and carried out a ChIP experiment to screen out the specific DNA sequence associated with geminin during somitogenesis. The final sequencing results for the DNA fragments bound to geminin suggested that part of intron 3 of* mib1* (Figure 4(a)) was associated with geminin. Furthermore, by mining the UCSC Genome Browser database, we found that, near the abovementioned geminin binding sequence, there exists a binding peak for H3K4me1 (but not for H3K4me3), indicating a possibility that intron 3 of* mib1* works as an enhancer of* mib1* transcription [68, 69]. To evaluate whether the interaction between geminin and intron 3 of* mib1* plays a crucial role in regulating* mib1 *transcription, we carried out luciferase reporter analysis* in vivo* [70]. We showed that partial intron 3 of* mib1* helped to enhance luciferase transcription (Figure 4(b), column 2). When geminin was downregulated in the embryos, the role of the enhancer was decreased (Figure 4(b), column 4), but overexpression of geminin can not increase the enhancer role of the intron 3 of Mib1 (Figure 4(b), column 3). These data indicated indirectly that geminin positively regulates* mib1 *transcription by binding to intron 3 of* mib1*. To further confirm the regulatory role of geminin in the expression of* mib1 in vivo*, we examined the expression of* mib*1 when geminin was knocked down. Expectedly,* mib1* transcription was downregulated in geminin morphants (Figure 4(c)). In addition, the activity of Notch signaling was greatly downregulated in* mib1* morphants, as significantly decreased expression levels of* her4* in the PSM, tail bud, and the forming somites were noted (Figure 4(e)). This result replicated previously reported findings in* mib1* mutant [17]. In* mib1* mutant, although Notch activity was downregulated, the expression of* deltaD* was upregulated (Figure S5C and S5F [17]) because lateral inhibition of Notch was reduced. Notably, in our study, the transcription of* deltaD* was downregulated in geminin morphants (Figures 1(g) and 3(b) and S5B and S5E). These data indicated that geminin positively regulates* mib1* transcription by binding to intron 3 of that gene, as well as the transcription of* deltaD* via another* mib1*-independent pathway.

### 3.5. Brg1 Facilitates Geminin Binding to Intron 3 of mib1 during the Regulation of mib1 Transcription

**Figure 5:**
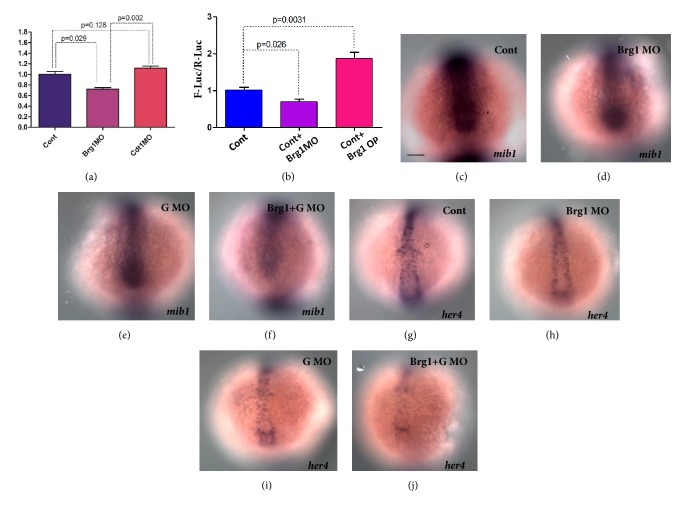
**Brg1 helps geminin to bind the intron of Mib1 during regulating Mib1 transcription**. (a, b) The ChIP experiments and the sequential qPCR showed that the quantity of intron 3 of Mib1 bound to geminin was decreased in* Brg1* morphants (a). The luciferase report analysis showed that down- and/or upregulating the activity of Brg1 decreased or increased the luciferase activity, respectively (b). (c-j) Compared with that in control morphants (c, d, 86.7% and 84.4%, n=30 and 32), the expressions of* Mib1* and* her4* were downregulated in* Brg1* morphants (d, h, 73.7% and 77.5%, n=38 and 40) and* geminin* morphants (e, i, 83.3% and 84.6%, n=36 and 39), respectively. Meanwhile in* Brg1* and* geminin* double morphants (f, j, 84.4% and 86.7%, n=32 and 30)* Mib1* and* her4* were downregulated strongly. Bar, 100*μ*M.

It has been reported that geminin regulates gene transcription in association with other cofactors [35, 58, 71]. During neurogenesis, geminin interacts with Brg1 to maintain the undifferentiated cell state by inhibiting interactions of Brg1 with the proneural basic helix-loop-helix gene [35]. Furthermore, Brg1 was reported to be associated with Baf60c to control Notch activity during LR asymmetry patterning in mouse and zebrafish [72]. Our previous study also showed that geminin is involved in LR asymmetry patterning [44]. These studies suggested a possibility that Brg1 may promote geminin binding to intron 3 of* mib1* to regulate* mib1* transcription. To evaluate this possibility, we employed ChIP and qPCR and revealed that when* brg1* was mildly knocked down, geminin bound intron 3 of Mib1 was mildly decreased (Figure 5(a), column 2). Since Cdt1/geminin and another DNA replication factors will form a complex to initiate DNA replication while not regulating genes transcription, our experiment also showed that geminin binding to intron 3 of* mib1* could be enhanced by blocking cdt1 translation (Figure 5(a), column 3). Furthermore, luciferase reporter analysis carried out in embryos showed that down- and/or upregulating* brg1* activity decreased or increased luciferase activity, respectively (Figure 5(b)), with the effects being similar to those seen in geminin morphants or geminin overexpression embryos (Figure 4(b)). To further evaluate the role of Brg1 in regulating* mib1* expression, we examined the expression of* mib1* in* brg1* morphants. The in situ experiments suggested that* mib1* transcription was mildly downregulated in embryos injected with* brg1MO* (Figure 5(d)). Moreover, this downregulation of* mib1* transcription was further strengthened by simultaneous downregulation of the activity of* brg1* and geminin (Figures 5(d)–5(f)), suggesting a synergistic role of geminin and Brg1 in regulating* mib1* expression. Because Notch activity was downregulated in both* mib1* mutant [17] and* mib1* morphant, to further evaluate the role of Brg1 in regulating* mib1* expression, we assessed the expression of* her4* in* brg1* morphants as well as in* brg1* and geminin double morphants. Our data demonstrated that downregulation of Brg1 resulted in decreased expression of* her4 *in both types of morphants (Figures 5(h)–5(j)). These results further suggested that Brg1 works as a coregulator of geminin in the positive modulation of Notch activity that, at least partially, is mediated by the effect of these proteins on* mib1* expression.

## 4. Discussion

The “clock-wavefront” model has been suggested to explain the mechanism of somite segmentation [2, 4], and many studies on mouse and zebrafish supported it. In this model, Notch signaling is essential for inducing oscillation of the expression of Notch effectors and final somite boundary formation [6, 8]. In addition, Fgf signaling, working as the wavefront gradient, contributes to precise positioning of the forming somite [21, 24]. Although there are presumably some links between these two processes during somitogenesis, how they are coordinated together has been rarely addressed. Here, we report that geminin regulates somite segmentation by orchestrating Notch and Fgf8 signaling pathways during somitogenesis.

In addition to the roles of geminin in neurogenesis, hematopoiesis, and gastrulation cell movement [37, 38, 42], our current study identified a novel role of geminin in somite formation. When geminin was knocked down, somites were defective, displaying the “shield” shape and blurry boundaries (Figures 1(b) and S1F, arrow). Notably, this phenotype was not because of cell apoptosis in geminin morphants, as the embryos still showed defective somites when cell apoptosis was prevented by coinjecting* p53MO* (Figures 1(c) and S1F, I). Further, our transplantation experiments substantiated this result further, showing the obscure somite boundary (Figure 1(j)) and delayed expression of somitic marker* deltaD *(Figure 1(g)) in the half sides of wild-type embryos that were transplanted with the cells from the donor embryos injected with* GemMO*.

**Figure 6:**
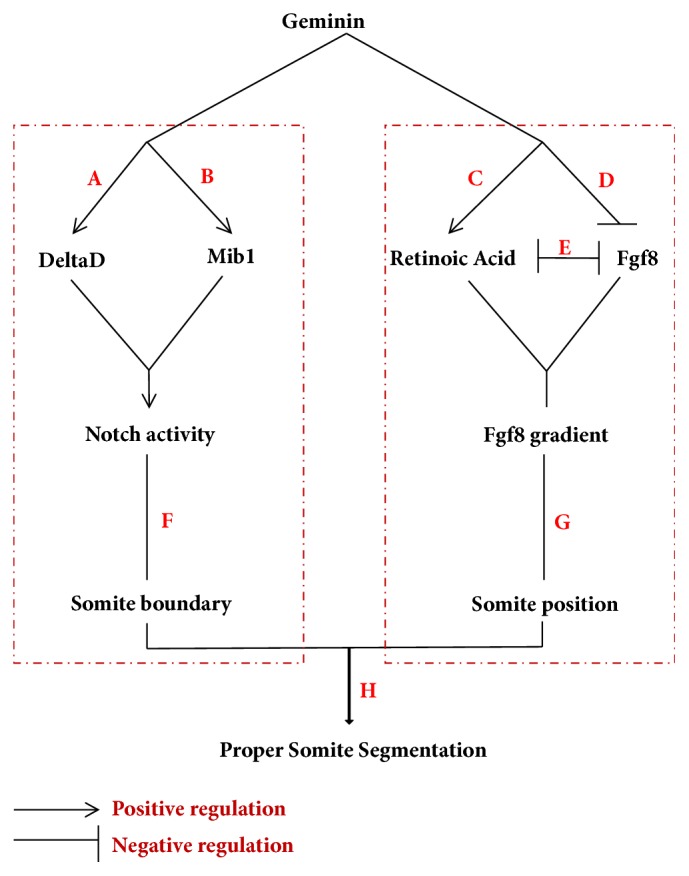
**The model for how geminin regulates somitogenesis**. During somitogenesis, geminin simultaneously regulates Notch activity (F) and Fgf8 signaling (G) to orchestrate proper somite segmentation (H). (A, B) Geminin positively regulates the transcription of Mib by binding to intron 3 of Mib (B); it also directly regulates the expression of DeltaD (A) and then collectively regulates Notch activity. On the other way, geminin negatively regulates the expression of fgf8 (D) but positively regulates the expression of raldh2 (retinoic acid) (C). RA and Fgf8 antagonize each other to form the* Fgf8* gradient retraction during somitogenesis (E).

Fgf signaling, working as the wavefront gradient, contributes to the position of somite segmentation in the anterior PSM. Disrupting the activity of Fgf8 resulted in somite size changes along the AP axis in mouse and zebrafish [21, 24]. Our data here showed increased expression of* fgf8* within the PSM in geminin morphants (Figures 2(b) and 6D), which likely led to narrower somites (Figure 1(h)). We also showed that the expression of* raldh2* decreased greatly (Figures 2(c) and 2(d)) in embryos with geminin loss of function, as were the antagonistic gradients of Fgf and RA in the PSM in zebrafish (Figures S2 and 6E). Thus, we could not determine which gene disruption was the primary reason of narrower somites in geminin morphants. It should be noted that* raldh2 *(Figure 2(c), arrow head) but not* fgf8* is expressed in heart progenitors, and* raldh2 *was greatly downregulated in this region (Figure 2(d), arrow head), suggesting a possibility that geminin loss of function initially downregulated RA signaling and then increased the expression of* fgf8* in the PSM.

Our results showed that Notch signaling was regulated by geminin (Figure 3(d)) and that Notch signaling partially mediated the effect of geminin on somite boundary formation (Figures 3(k) and 6F). As for the mechanism of this interaction, our ChIP experiments suggested that geminin, potentially in cooperation with Brg1, associates with intron 3 of* mib1 *(Figure 4(a)) and positively regulates* mib1 *transcription. Although geminin loss of function downregulated the expression of* deltaD *(Figure 3(b)) and activity of Notch signaling (Figure 3(d)) and resulted in somite polarity and sequential somite boundary defects (Figures 1(b), 1(i), 1(j), and 3(j)), the expression patterns of the oscillators* her1*,* her7*, and* deltaC* were normal (Figure S3A–F). These changes were different from those observed in embryos with other Notch-related mutations and in embryos treated with DAPT [50], in which the oscillators were expressed in the “salt and pepper” way. In addition, the expression levels of both* deltaD* and* mib1* were downregulated in geminin morphants, whereas the* deltaD* expression was previously found to be upregulated in* mib1* mutants [17] and, in the present study, in* mib1* morphants (Figures S4, C, and F). Thus, geminin positively regulates expression levels of* deltaD* and* mib1* and thereby affects Notch activity in a parallel way during somitogenesis.

G*eminin* morphants displayed downregulated Notch activity and disturbed somite boundary, but normal oscillation of cyclic genes (Figure S3A–F). Although this result was not consistent with observations in DAPT-treated embryos [50], it could be explained indirectly by some earlier studies. In a study that used approaches similar to ours,* foxc1a* loss of function blocked the formation of morphological somites along the whole AP axis and led to the downregulation of* notch5* and* notch6*, whereas oscillating expression pattern of* deltaC* and* deltaD* remained normal [73]. Recently, the new* mib1*-related mutant* mibnn*^*2002*^ was also shown to display early segmentation defect (from 7–10th somite stages), but without the disruption to the cyclic expression of the* deltaC *gene [74], which was different from the observations in another* mib* mutant that showed posterior somite defect and disturbed cyclic gene expression. These earlier reports indicated that the mechanism of oscillator regulation is complicated and that the downregulation Notch activity does not always lead to disrupted oscillation of cyclic gene expression. In our study, it was possible that geminin loss of function downregulated Notch activity through* mib1*-dependent and* mib1*-independent pathways (Figure 6A, B), and this downregulation was not as strong as that in Notch-related mutants or embryos treated with DAPT, in which the cyclic gene expression pattern was disturbed [50]. The regulation of the oscillation of cyclic gene expression is dose-dependent for Notch signaling, so the extent of Notch activity downregulation in geminin morphants may not have been strong enough to result in deficient oscillation of cyclic gene expression.

## 5. Conclusions

In summary, our data for the first time showed a critical role of geminin in regulating Fgf8 and Notch signaling during somitogenesis (Figure 6). On the one hand, in geminin morphants, the expression levels of* deltaD* and* mib1* were simultaneously downregulated (Figure 6A, B), concomitantly with the downregulation of Notch activity and consequential somite boundary defect (Figure 6F). This effect was likely mediated by the interaction of geminin, together with Brg1, with intron 3 of* mib1* and subsequent positive regulation of* mib1* transcription. On the other hand, geminin loss of function also resulted in the upregulation of* fgf8* and downregulation of* raldh2 via a direct or indirect way*, which led to delayed retraction of Fgf8 activity along the PSM to tail bud and resulted in the formation of narrower somites compared to those in control morphants. We conclude that geminin regulates Fgf8 and Notch signaling and thereby coordinates somite segmentation during somitogenesis.

## Data Availability

The data used to support the findings of this study are available from the corresponding author upon request.

## Supplementary Materials

Supplementary Materials
** Figure S1: geminin regulates somite formation in the early development. (A) At 5th somite stage, geminin is expressed in head, somite, PSM, and the tail bud. (B) The booked GemMO efficiently blocks the translation of geminin mRNA, and the caped-mRNA synthesized in vitro worked well**. (C) Compared with that in control morphants (A, n=22), the anterior-posterior (AP) axis is shortened in geminin morphants (84.3%, n=51) as well as in geminin and p53 double morphants (83.6%, n=55). (D) To compare with that in control morphants (n=24), the somite shape is deformed in geminin morphants (85.7%, n=49) as well as in geminin and p53 double morphants (86.8%, n=53). (E) In geminin morphants (83.3%, n=48), the cell apoptosis is more than control morphants (n=30). Meanwhile in geminin and p53 double morphants (I, 83%, n=47), the cell apoptosis could be rescued. Bar, 100*μ*M.** Figure S2: Fgf8 and RA signaling was regulated by geminin**. (A-D) When compared with that in control (A, C)* fgf8* expression was upregulated in* geminin* morphants (B, 88%, n=25), but the expression of* raldh2* was downregulated in geminin morphants (D, 90%, n=20). The expression of* tbx16l* was also upregulated in* geminin* morphants (F, 89.5%, n=19). Bar, 100*μ*M.** Figure S3: the activities of FGF and RA antagonize each other in the PSM**. (A, B) To compare with the expression of* raldhl2* in GFP negative control (A, 86%, n=43), raldhl2 was downregulated along AP after heat shock in transgene fish line Hsp70:dnFGFR1-GFP (B, 85.1%, n=47). (C, D) Compared with that in DMSO control (C, 88.9%, n=45), fgf8 was upregulated along AP after dealing with BMS (D, 84%, n=50). Bar, 100*μ*M.** Figure S4: Notch activity in geminin morphants and the role of Notch in somitogenesis**. (A, B) To compare with the expression of* DeltaC* in control (A, 88.3%, n=43), deltaC was downregulated in geminin morphants (B, 85.1%, n=47). The deltaC in new forming somite displayed wild “salt and pepper” way (A, B, arrow showed). (C-F) The downstream genes of Notch signal her1 and her7 were also slightly downregulated in the embryos injected with GemMO (D and F, 87.2% and 86.9%, n=47 and n=46), while the expression of her1 and her7 was normal in control embryos (C and E, 84.3% and 85.3%, n=32 and 41). (G-K) Comparing with that in control (G and J, 86.2% and 88.2%, n=29 and 34),*mespaa* and anterior polarity* papc* were decreased in geminin morphants (H and K, 80% and 82.7%, n=30 and 29). Bar, 100*μ*M.** Figure S5: the expression of deltaD is different in geminin morphants and mib1 morphants**. (A-F) To compare with the expression of* deltaD* in control (A and D, 85%, n=40),* deltaD* was downregulated in geminin morphants (B and E, 86.3%, n=44) and upregulated in mib1 morphants (C and F, 81.8%, n=33). Bar, 100*μ*M.
