# Supplementary figures and images for: Geminin Orchestrates Somite Formation by Regulating Fgf8 and Notch Signaling
Source: Biomed Res Int. 2018 Jun 7;2018:6543196. doi: 10.1155/2018/6543196 (PMC6011172; doi:10.1155/2018/6543196)

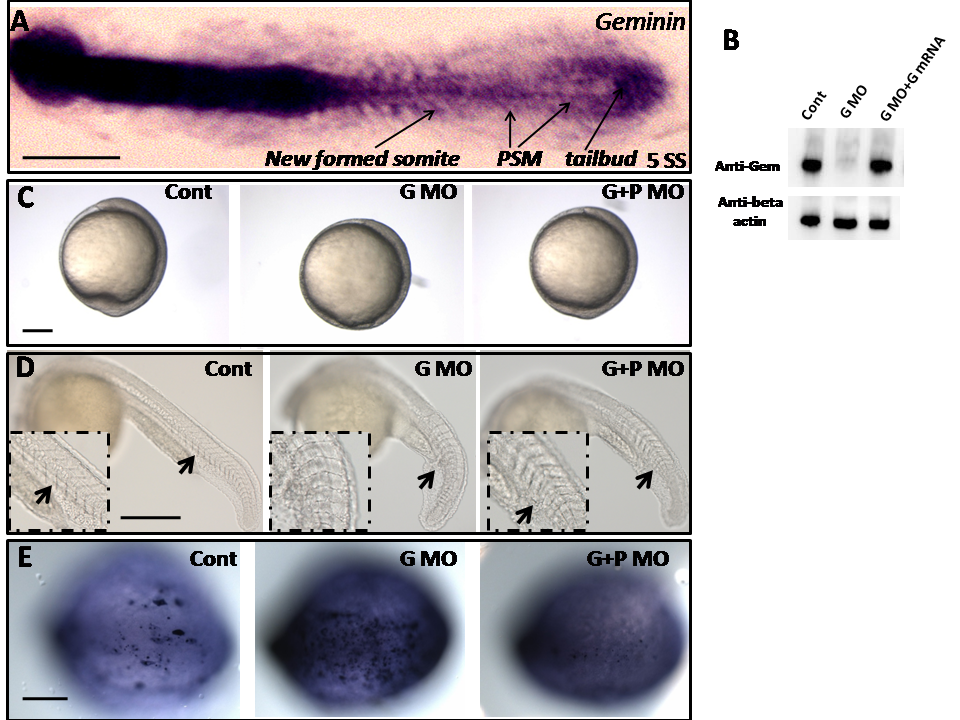

Supplement: Supplementary Materials — Figure S1: geminin regulates somite formation in the early development. (A) At 5th somite stage, geminin is expressed in head, somite, PSM, and the tail bud. (B) The booked GemMO efficiently blocks the translation of geminin mRNA, and the caped-mRNA synthesized in vitro worked well. (C) Compared with that in control morphants (A, n=22), the anterior-posterior (AP) axis is shortened in geminin morphants (84.3%, n=51) as well as in geminin and p53 double morphants (83.6%, n=55). (D) To compare with that in control morphants (n=24), the somite shape is deformed in geminin morphants (85.7%, n=49) as well as in geminin and p53 double morphants (86.8%, n=53). (E) In geminin morphants (83.3%, n=48), the cell apoptosis is more than control morphants (n=30). Meanwhile in geminin and p53 double morphants (I, 83%, n=47), the cell apoptosis could be rescued. Bar, 100μM. Figure S2: Fgf8 and RA signaling was regulated by geminin. (A-D) When compared with that in control (A, C) fgf8 expression was upregulated in geminin morphants (B, 88%, n=25), but the expression of raldh2 was downregulated in geminin morphants (D, 90%, n=20). The expression of tbx16l was also upregulated in geminin morphants (F, 89.5%, n=19). Bar, 100μM. Figure S3: the activities of FGF and RA antagonize each other in the PSM. (A, B) To compare with the expression of raldhl2 in GFP negative control (A, 86%, n=43), raldhl2 was downregulated along AP after heat shock in transgene fish line Hsp70:dnFGFR1-GFP (B, 85.1%, n=47). (C, D) Compared with that in DMSO control (C, 88.9%, n=45), fgf8 was upregulated along AP after dealing with BMS (D, 84%, n=50). Bar, 100μM. Figure S4: Notch activity in geminin morphants and the role of Notch in somitogenesis. (A, B) To compare with the expression of DeltaC in control (A, 88.3%, n=43), deltaC was downregulated in geminin morphants (B, 85.1%, n=47). The deltaC in new forming somite displayed wild “salt and pepper” way (A, B, arrow showed). (C-F) The downstream genes of Notch s [file 6543196.f1.zip › Fig. S1.TIF]

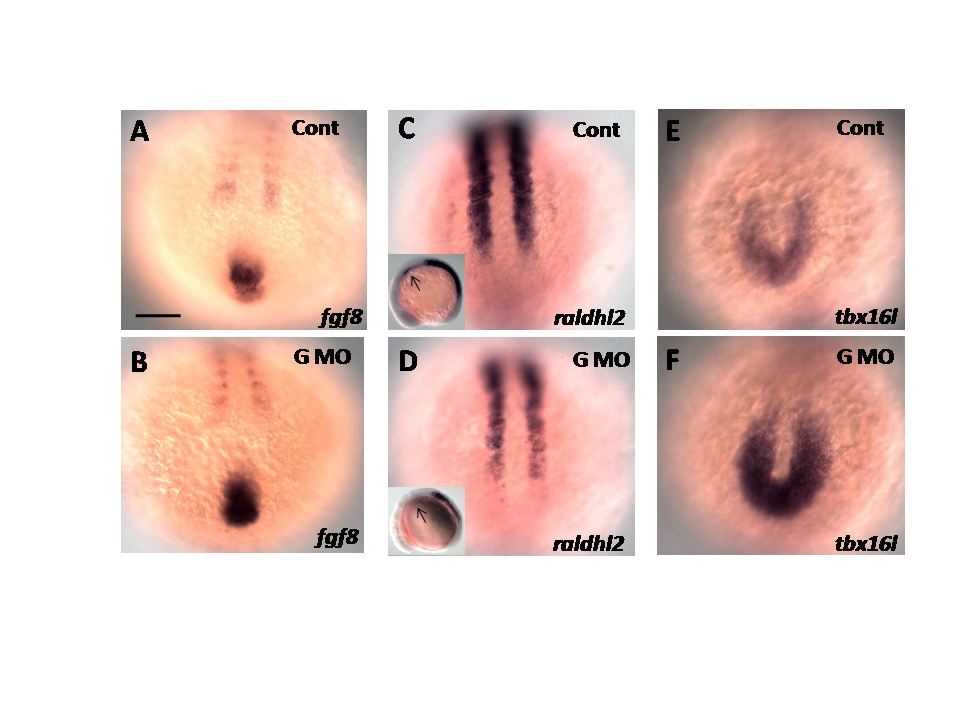

Supplement: Supplementary Materials — Figure S1: geminin regulates somite formation in the early development. (A) At 5th somite stage, geminin is expressed in head, somite, PSM, and the tail bud. (B) The booked GemMO efficiently blocks the translation of geminin mRNA, and the caped-mRNA synthesized in vitro worked well. (C) Compared with that in control morphants (A, n=22), the anterior-posterior (AP) axis is shortened in geminin morphants (84.3%, n=51) as well as in geminin and p53 double morphants (83.6%, n=55). (D) To compare with that in control morphants (n=24), the somite shape is deformed in geminin morphants (85.7%, n=49) as well as in geminin and p53 double morphants (86.8%, n=53). (E) In geminin morphants (83.3%, n=48), the cell apoptosis is more than control morphants (n=30). Meanwhile in geminin and p53 double morphants (I, 83%, n=47), the cell apoptosis could be rescued. Bar, 100μM. Figure S2: Fgf8 and RA signaling was regulated by geminin. (A-D) When compared with that in control (A, C) fgf8 expression was upregulated in geminin morphants (B, 88%, n=25), but the expression of raldh2 was downregulated in geminin morphants (D, 90%, n=20). The expression of tbx16l was also upregulated in geminin morphants (F, 89.5%, n=19). Bar, 100μM. Figure S3: the activities of FGF and RA antagonize each other in the PSM. (A, B) To compare with the expression of raldhl2 in GFP negative control (A, 86%, n=43), raldhl2 was downregulated along AP after heat shock in transgene fish line Hsp70:dnFGFR1-GFP (B, 85.1%, n=47). (C, D) Compared with that in DMSO control (C, 88.9%, n=45), fgf8 was upregulated along AP after dealing with BMS (D, 84%, n=50). Bar, 100μM. Figure S4: Notch activity in geminin morphants and the role of Notch in somitogenesis. (A, B) To compare with the expression of DeltaC in control (A, 88.3%, n=43), deltaC was downregulated in geminin morphants (B, 85.1%, n=47). The deltaC in new forming somite displayed wild “salt and pepper” way (A, B, arrow showed). (C-F) The downstream genes of Notch s [file 6543196.f1.zip › Fig. S2.TIF]

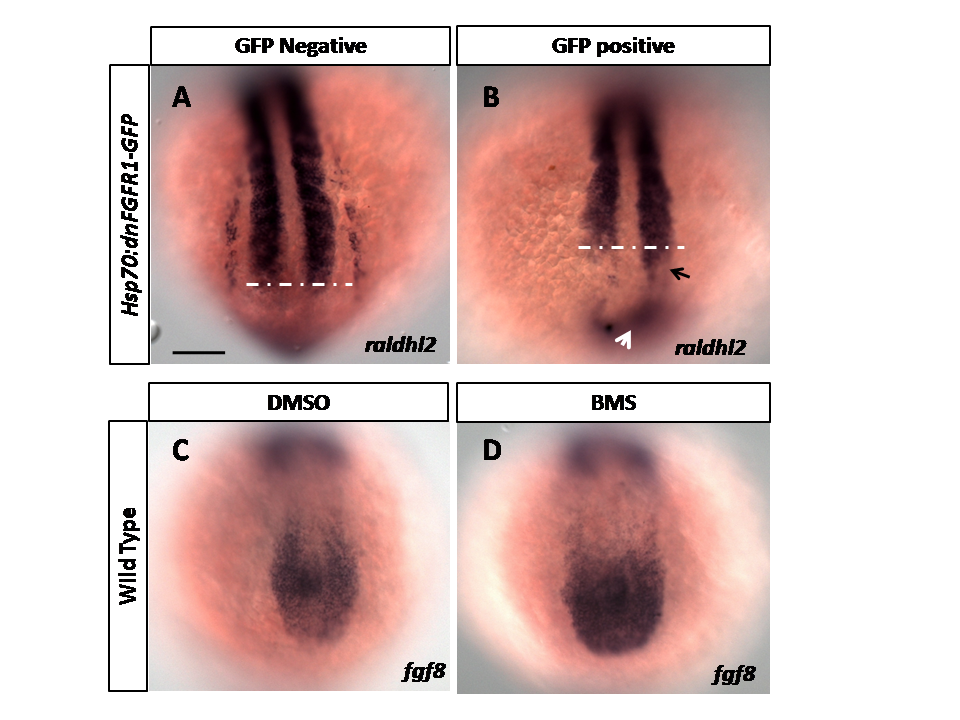

Supplement: Supplementary Materials — Figure S1: geminin regulates somite formation in the early development. (A) At 5th somite stage, geminin is expressed in head, somite, PSM, and the tail bud. (B) The booked GemMO efficiently blocks the translation of geminin mRNA, and the caped-mRNA synthesized in vitro worked well. (C) Compared with that in control morphants (A, n=22), the anterior-posterior (AP) axis is shortened in geminin morphants (84.3%, n=51) as well as in geminin and p53 double morphants (83.6%, n=55). (D) To compare with that in control morphants (n=24), the somite shape is deformed in geminin morphants (85.7%, n=49) as well as in geminin and p53 double morphants (86.8%, n=53). (E) In geminin morphants (83.3%, n=48), the cell apoptosis is more than control morphants (n=30). Meanwhile in geminin and p53 double morphants (I, 83%, n=47), the cell apoptosis could be rescued. Bar, 100μM. Figure S2: Fgf8 and RA signaling was regulated by geminin. (A-D) When compared with that in control (A, C) fgf8 expression was upregulated in geminin morphants (B, 88%, n=25), but the expression of raldh2 was downregulated in geminin morphants (D, 90%, n=20). The expression of tbx16l was also upregulated in geminin morphants (F, 89.5%, n=19). Bar, 100μM. Figure S3: the activities of FGF and RA antagonize each other in the PSM. (A, B) To compare with the expression of raldhl2 in GFP negative control (A, 86%, n=43), raldhl2 was downregulated along AP after heat shock in transgene fish line Hsp70:dnFGFR1-GFP (B, 85.1%, n=47). (C, D) Compared with that in DMSO control (C, 88.9%, n=45), fgf8 was upregulated along AP after dealing with BMS (D, 84%, n=50). Bar, 100μM. Figure S4: Notch activity in geminin morphants and the role of Notch in somitogenesis. (A, B) To compare with the expression of DeltaC in control (A, 88.3%, n=43), deltaC was downregulated in geminin morphants (B, 85.1%, n=47). The deltaC in new forming somite displayed wild “salt and pepper” way (A, B, arrow showed). (C-F) The downstream genes of Notch s [file 6543196.f1.zip › Fig. S3.TIF]

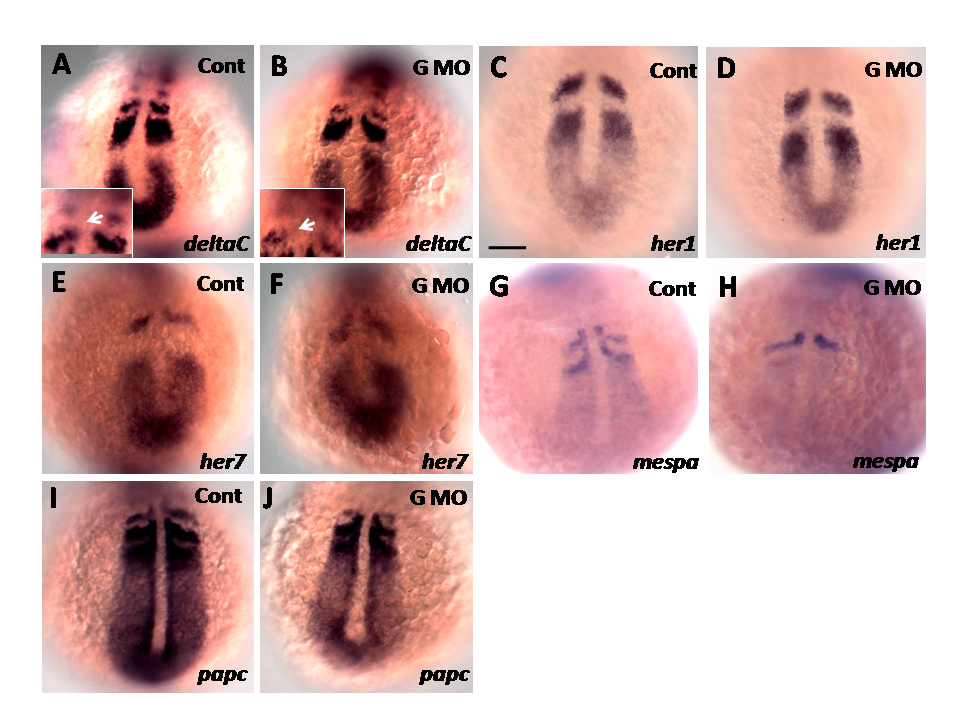

Supplement: Supplementary Materials — Figure S1: geminin regulates somite formation in the early development. (A) At 5th somite stage, geminin is expressed in head, somite, PSM, and the tail bud. (B) The booked GemMO efficiently blocks the translation of geminin mRNA, and the caped-mRNA synthesized in vitro worked well. (C) Compared with that in control morphants (A, n=22), the anterior-posterior (AP) axis is shortened in geminin morphants (84.3%, n=51) as well as in geminin and p53 double morphants (83.6%, n=55). (D) To compare with that in control morphants (n=24), the somite shape is deformed in geminin morphants (85.7%, n=49) as well as in geminin and p53 double morphants (86.8%, n=53). (E) In geminin morphants (83.3%, n=48), the cell apoptosis is more than control morphants (n=30). Meanwhile in geminin and p53 double morphants (I, 83%, n=47), the cell apoptosis could be rescued. Bar, 100μM. Figure S2: Fgf8 and RA signaling was regulated by geminin. (A-D) When compared with that in control (A, C) fgf8 expression was upregulated in geminin morphants (B, 88%, n=25), but the expression of raldh2 was downregulated in geminin morphants (D, 90%, n=20). The expression of tbx16l was also upregulated in geminin morphants (F, 89.5%, n=19). Bar, 100μM. Figure S3: the activities of FGF and RA antagonize each other in the PSM. (A, B) To compare with the expression of raldhl2 in GFP negative control (A, 86%, n=43), raldhl2 was downregulated along AP after heat shock in transgene fish line Hsp70:dnFGFR1-GFP (B, 85.1%, n=47). (C, D) Compared with that in DMSO control (C, 88.9%, n=45), fgf8 was upregulated along AP after dealing with BMS (D, 84%, n=50). Bar, 100μM. Figure S4: Notch activity in geminin morphants and the role of Notch in somitogenesis. (A, B) To compare with the expression of DeltaC in control (A, 88.3%, n=43), deltaC was downregulated in geminin morphants (B, 85.1%, n=47). The deltaC in new forming somite displayed wild “salt and pepper” way (A, B, arrow showed). (C-F) The downstream genes of Notch s [file 6543196.f1.zip › Fig. S4.TIF]

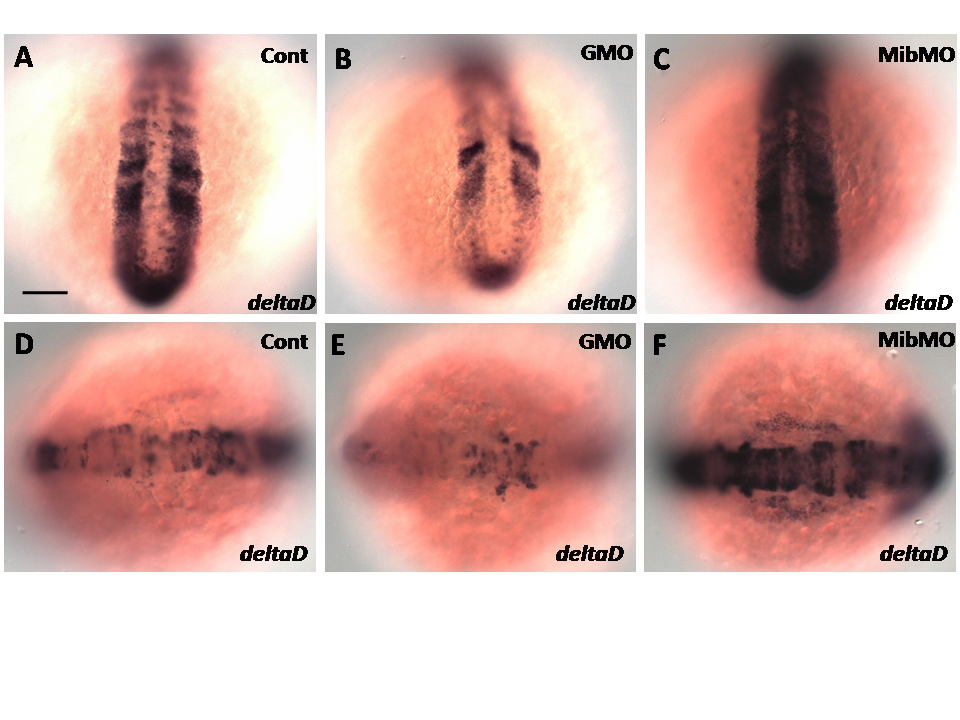

Supplement: Supplementary Materials — Figure S1: geminin regulates somite formation in the early development. (A) At 5th somite stage, geminin is expressed in head, somite, PSM, and the tail bud. (B) The booked GemMO efficiently blocks the translation of geminin mRNA, and the caped-mRNA synthesized in vitro worked well. (C) Compared with that in control morphants (A, n=22), the anterior-posterior (AP) axis is shortened in geminin morphants (84.3%, n=51) as well as in geminin and p53 double morphants (83.6%, n=55). (D) To compare with that in control morphants (n=24), the somite shape is deformed in geminin morphants (85.7%, n=49) as well as in geminin and p53 double morphants (86.8%, n=53). (E) In geminin morphants (83.3%, n=48), the cell apoptosis is more than control morphants (n=30). Meanwhile in geminin and p53 double morphants (I, 83%, n=47), the cell apoptosis could be rescued. Bar, 100μM. Figure S2: Fgf8 and RA signaling was regulated by geminin. (A-D) When compared with that in control (A, C) fgf8 expression was upregulated in geminin morphants (B, 88%, n=25), but the expression of raldh2 was downregulated in geminin morphants (D, 90%, n=20). The expression of tbx16l was also upregulated in geminin morphants (F, 89.5%, n=19). Bar, 100μM. Figure S3: the activities of FGF and RA antagonize each other in the PSM. (A, B) To compare with the expression of raldhl2 in GFP negative control (A, 86%, n=43), raldhl2 was downregulated along AP after heat shock in transgene fish line Hsp70:dnFGFR1-GFP (B, 85.1%, n=47). (C, D) Compared with that in DMSO control (C, 88.9%, n=45), fgf8 was upregulated along AP after dealing with BMS (D, 84%, n=50). Bar, 100μM. Figure S4: Notch activity in geminin morphants and the role of Notch in somitogenesis. (A, B) To compare with the expression of DeltaC in control (A, 88.3%, n=43), deltaC was downregulated in geminin morphants (B, 85.1%, n=47). The deltaC in new forming somite displayed wild “salt and pepper” way (A, B, arrow showed). (C-F) The downstream genes of Notch s [file 6543196.f1.zip › Fig. S5.TIF]
